# *De novo* achondroplasia causing four consecutive unsuccessful pregnancies: a case report

**DOI:** 10.1186/1752-1947-6-256

**Published:** 2012-08-30

**Authors:** Anthony Osita Igwegbe, George Uchenna Eleje, Ikechukwu Francis Ugwueke

**Affiliations:** 1 Department of Obstetrics and Gynaecology, Nnamdi Azikiwe University Teaching Hospital, PMB 5025, Nnewi, Anambra State, Nigeria

## Abstract

**Introduction:**

The incidence of achondroplasia is very low, and the birth of two or more consecutive babies with achondroplasia to unaffected parents is a rarity. We report a rare case of recurrent achondroplasia in babies of unaffected parents.

**Case presentation:**

A 29-year-old Nigerian Igbo woman who has had three consecutive dead achondroplastic babies presented at a gestational age of 31 weeks with a two-hour history of drainage of liquor and vaginal bleeding. Neither she nor her husband had features of achondroplasia. Fundal height was compatible with the gestational age. Fetal heart activity was present. An abdominal ultrasound examination showed a viable fetus with short long bones, oligohydramnios, and a fundal placenta with a small retroplacental blood clot. Our patient was stabilized and had an emergency Cesarean section for grade 1 abruptio placentae. A live male baby with Apgar scores of 4 at one minute and 5 at 10 minutes was delivered. The baby had classic features of achondroplasia and died shortly after birth.

**Conclusions:**

To the best of our knowledge, this is the first reported case of recurrent achondroplasia in siblings of unaffected parents in Nigeria. Management is challenging, and the outcomes of future pregnancies appear bleak. However, proper counseling and follow-up are needed. There is also a need to establish preconception clinics and facilities for prenatal genetic diagnosis and gene therapy in developing countries.

## Introduction

Achondroplasia is a genetic disorder of bone growth and usually is evident at birth. It affects about one in 10,000 to one in 30,000 births and occurs in all races and in both sexes [1]. It is the most common of a group of growth defects characterized by abnormal body proportions: short stature with disproportionate short limbs [1,2].

It is caused by a mutation in a gene, fibroblast growth factor receptor 3 (*FGFR3*), that is located in chromosome 4 (chromosomal locus 4p16.3), which is one of the physiological regulators of linear bone growth [3,4]. The mutation leads to excessive tyrosine kinase activity in the receptors within the cartilaginous growth plate, thereby inhibiting bone growth [5]. All bones that form by endochondral ossification are affected, but bones that form by membranous ossification are not affected, thus allowing the skull vault to develop normally [3,4].

It is inherited as a Mendelian autosomal dominant trait with complete penetrance; all individuals with the trait must have achondroplasia [3-5]. Inheritance of one abnormal gene from each parent results in severe skeletal abnormalities that may lead to early death (in the fatal form of the disease). Parents of children with achondroplasia resulting from a new mutation usually are of normal height. Typically, these parents have no other children with achondroplasia, and the chances of their having a second affected child are less than 1% [6,7].

In more than 80% of cases, achondroplasia is not inherited but results from a new or *de novo* dominant mutation that occurs in the egg or sperm cell that forms the embryo with a mutation rate estimated to be 0.000014 per gamete per generation [2,5]. A reoccurrence of this, especially in a non-achondroplastic couple, is a rare and perplexing event, and the outcome of future pregnancies appears bleak, as in the case reported here.

## Case presentation

A 29-year-old, Nigerian Igbo woman who had not pre-registered at a hospital and who was in her fourth pregnancy presented at the labor ward at a gestational age of 31 weeks with a two-hour history of spontaneous drainage of liquor and subsequent vaginal bleeding. She has had three consecutive deliveries of dead achondroplastic babies and had no living child. The vaginal bleeding was moderate, and there was no dizziness or fainting spells. There was no history of bleeding from other sites, abdominal pain, trauma, fever, dysuria, vaginal discharge, or chronic cough. She was not known to be hypertensive or diabetic. She had no history of surgery or radiation exposure. There was no family history of achondroplasia.

The index pregnancy was spontaneously conceived and was supported by her husband, who was a 34-year-old trader. She booked at a gestational age of 13 weeks in a private hospital and her booking parameters were said to be normal. There was no previous ultrasound examination, and the pregnancy had been uneventful until presentation. She was referred to the teaching hospital because of the spontaneous rupture of membranes (SROM) and vaginal bleeding.

Her first confinement was in 2005. She had SROM at a gestational age of 30 weeks and delivered a live achondroplastic female baby in a private hospital. However, the baby died a few minutes after delivery.

Her second confinement was in January 2008. She had ultrasound diagnoses of gross fetal abnormality and achondroplasia in a private hospital. The pregnancy was consequently terminated by using intra-vaginal misoprostol at a gestational age of 25 weeks.

The third confinement was in January 2009. She booked at the teaching hospital at a gestational age of 28 weeks. Her hemoglobin genotype is AA and her blood group is O Rhesus D-positive. The result of a veneral disease research laboratory test was non-reactive. The results of a test for human immunodeficiency virus I and II were negative. The results of fasting blood sugar and two-hour postprandial tests were normal. She received one course each of intermittent preventive therapy for malaria and tetanus toxoid at 28 weeks of gestation. An abdominal ultrasonography scan at 30 weeks revealed features of fetal achondroplasia and polyhydramnios. She had a premature rupture of membranes at 31 weeks of gestation. She had an intra-uterine fetal death while on conservative management and subsequently a spontaneous vertex delivery of a fresh stillborn boy that weighed 1.2kg. The following features were noted: short upper and lower limbs, a relatively large head circumference, a conjoined and patent anterior fontanelle, and a gangrenous intestine. An anatomical diagnosis of achondroplasia was made at autopsy.

A clinical examination revealed a woman who was in painful distress, afebrile, and anicteric but pale. There was no pedal edema. Her height was 1.68m and her body weight was 60kg. The height of her husband was 1.70m. Neither had any features of achondroplasia. Her abdomen was enlarged and moved with respiration. There was generalized tenderness in her abdomen. The symphysiofundal height was 28cm. The abdomen was hard, and the fetal parts were not easily discernible. The fetal heart rate was 160 beats per minute.

The urgent packed cell volume was 30%, and the results of urine analysis and clotting time were normal. The transabdominal ultrasound examination at the labor ward revealed oligohydramnios and a viable fetus with achondroplasia. The placenta was fundal with small retroplacental blood collection. A diagnosis of abruptio placentae grade 1 was made. Our patient and her husband were counseled on the diagnosis and options of delivery. They consented to delivery via Cesarean section.

The intra-operative findings included an achondroplastic live male baby who had Apgar scores of 4 at one minute and 5 at 10 minutes and who weighed 1.1kg. The baby had a relatively large head circumference, normal length of trunk, short limbs, and broad hands and feet (rhizomelic) as well as a protruding tongue, a large defect joining anterior and posterior fontanelles, hepatosplenomegaly, and low-set ears. However, the baby died 30 minutes after birth. The estimated blood loss was 400mL, excluding a retroplacental clot of about 100mL. Her post-operative recovery was satisfactory. A diagnosis of recurrent achondroplasia was made.

## Discussion

This is the first reported case of *de novo* achondroplasia, which is the most common of the human chondrodysplasias, in our center [6]. Few cases of recurrent sporadic (*de novo*) achondroplasia have been reported [7,8]. The term was coined over a century ago to distinguish individuals with a disproportionate short stature from individuals with a proportionate short stature.

The pathophysiology of the genetic mutation for *de novo* achondroplasia remains hypothetical. Fryns *et al*. [9] had suggested germinal mosaicism, and Opitz [10] suggested unstable premutation with reduced penetrance. A study has also shown that advanced paternal age, particularly over 35 years, strongly correlates with achondroplasia and certain other autosomal dominant conditions caused by new mutations [11]. This was not applicable in the reported case since both parents were younger than 35 years old.

Prenatal genetic diagnosis may be made through amniocentesis and a chorionic villus sample. Second trimester ultrasonography also may be diagnostic. The ultrasound features of fetal achondroplasia include a femur length in less than the fifth centile with biparietal diameter, abdominal circumference and foot length within normal limits, a femur-to-foot length ratio of less than 0.87, and a femur length-to-abdominal circumference ratio of less than 0.18. In high-risk couples, a combination of ultrasongraphy and genetic studies would establish an accurate diagnosis, which is required for appropriate counseling [12]. A misdiagnosis chance of 7.8%, based on sonographic biometric data, has been reported [13]. Owing to the routine prenatal ultrasound examination in developed countries, achondroplasia of many affected fetuses is recognized in the third trimester of pregnancy and so families can be prepared for the birth of an affected child.

The features of achondroplasia can easily be identified clinically and radiologically at birth and infancy [6,7]. The rhizomelic appearance is pathognomonic [6]. Features include a long narrow trunk with very short limbs, macrocephaly with frontal bossing, hyperextensible joints, abducted hip, moderate hypotonia, and delayed milestones. The radiological features include a large cranium with a protuberant forehead, short long bones, and a V-shaped appearance of distal femoral epiphyses. However, for about 20% of affected individuals, achondroplasia may not be recognized at birth [2]. In doubtful cases, genetic testing using a blood sample may be done. In the case reported, diagnosis was made by using the physical features and autopsy result of the previous baby. Genetic studies could be diagnostic and therapeutic. However, such facilities are not available in our center.

The management of recurrent achondroplasia is more difficult and challenging in resource-poor countries. This is because of the unavailability of tools for genetic diagnosis and therapy. Human growth hormone and osteotomies (surgical limb lengthening) had been used with very limited success to increase stature in children and are no longer recommended [7].

Some recent therapeutic innovations are based on the pathogenesis of achondroplasia. With emerging knowledge of the molecular pathogenesis, interest has begun to shift to therapy intended to counter the effects of the overactive receptor. Although the precise mechanism is not known, the stabilization of FGFR3 dimerization caused by the mutation is believed to enhance the kinase activity of the receptor. Strategies involve inhibition of the FGFR3 receptor activity by using chemical agents or receptor-blocking antibodies [14]. These agents would downregulate the excessive tyrosine kinase activity of the receptors. Another molecular therapy involves C-type natiuretic peptide (CNP), which because of its effects on fluid and electrolyte balance and vascular tone is being evaluated as a therapeutic agent for certain cardiovascular diseases. Since CNP has recently been shown to downregulate fibroblast growth factor-induced activation of mitogen-activated protein kinase signaling pathways in growth plate chondrocytes and to counteract the effects of the achondroplasia mutation in mice, it has been suggested as a possible treatment for achondroplasia in humans [15].

All of these therapeutic approaches are in the early stages of development. However, they are promising and will reach the clinical trial phase of development. In developing countries where gene therapy is not available, donor insemination, ovum donation, and child adoption may be options for the management of recurrent *de novo* achondroplasia.

## Conclusions

This presentation highlights one of the genetic problems that could cause recurrent pregnancy loss and dilemmas in management in our environment. In spite of the emphasis on reducing maternal mortality and infections, there is also a need to establish preconception clinics and facilities for prenatal genetic diagnosis and gene therapy in developing countries.

## Consent

Written informed consent was obtained from the patient for publication of this case report and any accompanying images. A copy of the written consent is available for review by the Editor-in-Chief of this journal.

## Abbreviations

CNP: C-type natiuretic peptide; FGFR3: Fibroblast growth factor receptor 3; SROM: Spontaneous rupture of membranes.

## Competing interests

The authors declare that they have no competing interests.

## Authors’ contributions

AOI performed the surgery, assisted in the writing of the manuscript and in the obstetrics work-up of the patient, and helped to critically revise the manuscript. GUE worked on sending the specimen for pathologic analysis, assisted in the drafting of the manuscript, performed PubMed research, and helped to critically revise the manuscript. IFU performed the obstetrics work-up of the patient, assisted in the writing of the manuscript, and performed PubMed research. All authors read and approved the final manuscript.
